# Renal sinus leiomyoma mimicking papillary renal cell carcinoma: A case report

**DOI:** 10.1016/j.radcr.2026.01.092

**Published:** 2026-03-05

**Authors:** Shinichi Ozaki, Takahiro Yamamoto, Sou Unten, Ryotaro Shimizu, Nobuo Yokoyama, Ayumi Asai, Akiko Narita, Hiroaki Okada, Masashi Shimohira, Haruka Kurosu, Takanori Ito, Kojiro Suzuki

**Affiliations:** a Department of Radiology, Aichi Medical University Hospital, Nagakute, Aichi, Japan; b Department of Urology, Aichi Medical University Hospital, Nagakute, Aichi, Japan; c Department of Pathology, Aichi Medical University Hospital, Nagakute, Aichi, Japan

**Keywords:** Renal leiomyoma, Papillary renal cell carcinoma, Renal sinus, Imaging characteristics, CT, MRI

## Abstract

Renal leiomyomas are rare benign tumors that predominantly affect women in their 40s and 50s. Here, we report a case of renal leiomyoma originating from the renal sinus of a woman in her 30s. The lesion was incidentally discovered on abdominal magnetic resonance imaging as a 4 cm, well circumscribed mass with smooth margins located in the right renal sinus. Dynamic contrast-enhanced computed tomography (CT) revealed homogenous and progressive enhancement, while T2-weighted magnetic resonance imaging (MRI) demonstrated uniformly low signal. Although imaging findings suggested a renal sinus tumor, papillary renal carcinoma could not be definitively excluded by imaging. Due to the difficulty in performing computed tomography-guided biopsy, a nephrectomy was undertaken. Pathological examination confirmed the diagnosis of a renal leiomyoma originating from the renal sinus.

## Introduction

Renal leiomyomas are rare tumors, accounting for only 0.3% of all renal tumors, and occur predominantly in women in their 40s and 50s [1]. These tumors typically originate from the subcapsular or capsular regions of the kidney, although involvement of the renal sinus is uncommon [2,3]. Because they are benign, their differentiation from malignant tumors on imaging is challenging. Herein, we present a case of a renal sinus leiomyoma in a woman in her 30s, in which imaging findings closely resembled those of papillary renal cell carcinoma. As a result, the possibility of leiomyoma was not considered, leading to a nephrectomy. A literature review indicates that accurate differentiation from papillary renal carcinoma requires careful assessment of both epidemiological factors and the tumor's anatomical origin.

## Case report

The patient was a female in her 30s who presented with the chief complaint of a right kidney tumor. An abdominal ultrasound during a physical examination revealed a slight enlargement of the main pancreatic duct. Abdominal magnetic resonance imaging (MRI), performed for further examination, no significant dilation of the main pancreatic duct was observed, but incidentally revealed a tumor in the right kidney. No other abnormalities were observed on physical examination. The patient had a history of sleeve gastrectomy for obesity, and both blood and urine test results were within normal limits.

**Fig. 1:**
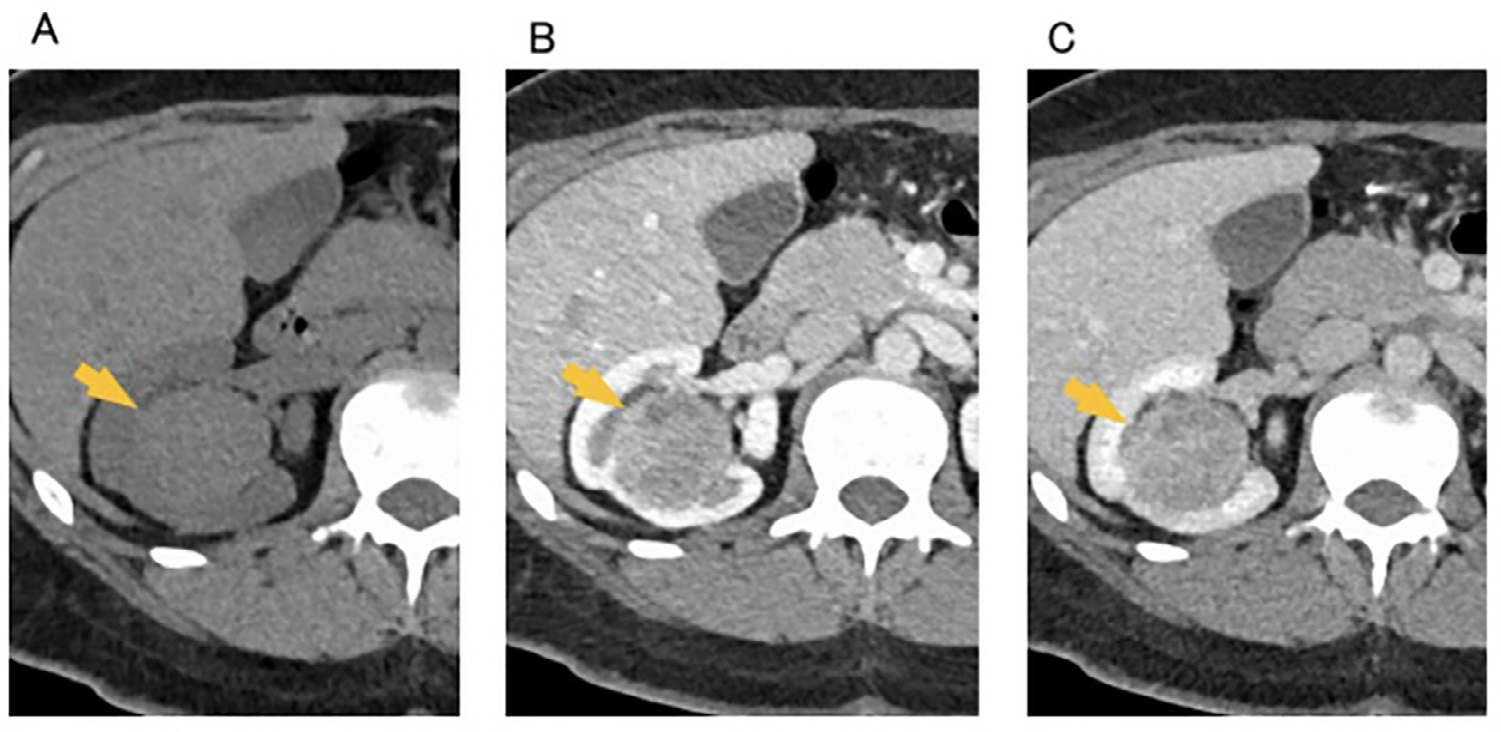
Dynamic contrast-enhanced CT. (A) unenhanced, (B) corticomedullary phase, and (C) nephrogenic phase. There is a 4 cm × 3.5 cm × 4 cm tumor with smooth margins and well-defined borders in the right renal sinus (yellow arrow). On unenhanced CT, the tumor appears to have a homogeneous density of 46 HU (A). Dynamic contrast-enhanced imaging demonstrates relatively uniform and gradual enhancement, with 73 HU in the corticomedullary phase and 104 HU in the nephrogenic phase (B, C).

**Fig. 2:**
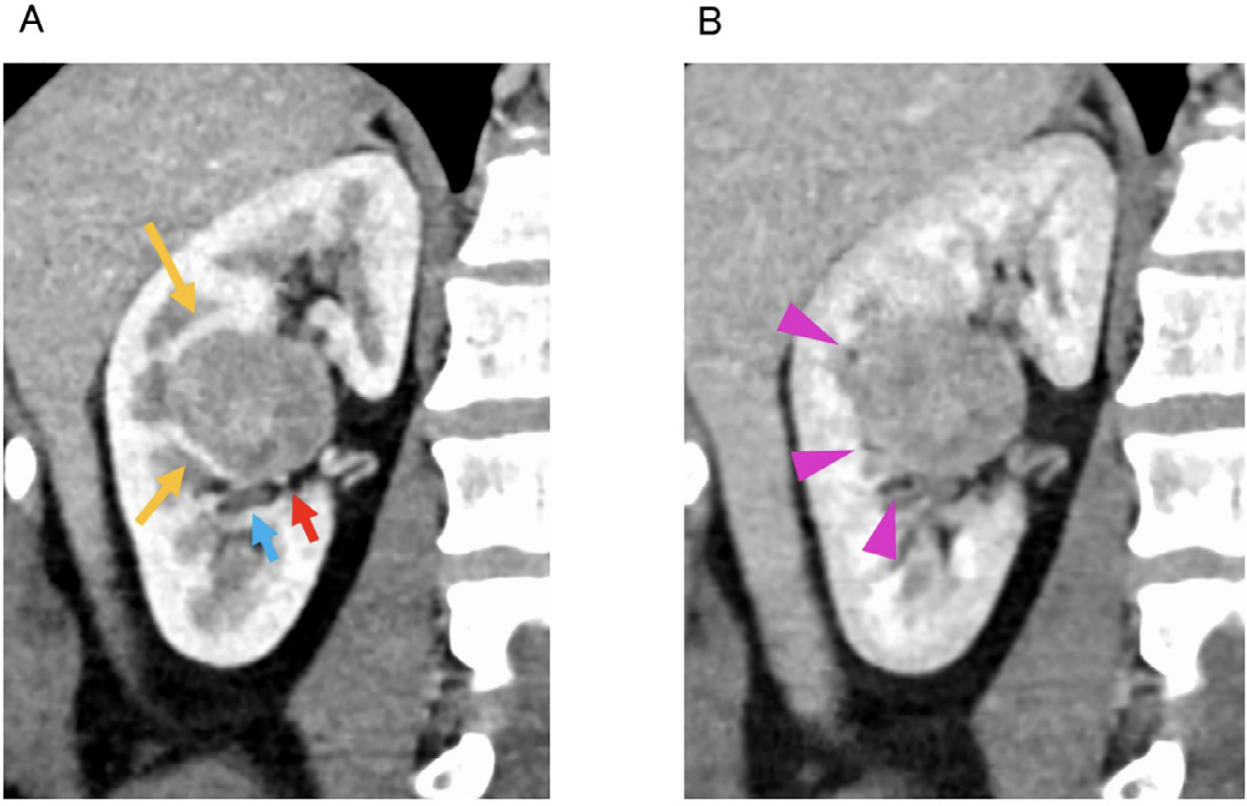
Coronal image of dynamic contrast-enhanced CT. (A) corticomedullary phase, and (B) nephrogenic phase. Tumor is compressing the renal parenchyma (yellow arrow). Vessels (red arrow), calyces (light blue arrow), and small fat (pink arrowhead) are present in multiple locations between the tumor and the renal parenchyma.

Dynamic contrast-enhanced computed tomography (CT) and MRI were performed at our hospital. CT imaging revealed a 4 cm × 3.5 cm × 4 cm mass with smooth margins located in the right renal sinus (Fig. 1). On unenhanced CT, the tumor appeared homogeneous with a slightly higher density compared with the renal parenchyma. On dynamic contrast-enhanced CT, it demonstrated a relatively homogeneous enhancement that gradually increase. Coronal dynamic contrast-enhanced CT images revealed that the tumor was compressing the renal parenchyma (Fig. 2). There were renal calyces, vessels, and multiple areas of renal sinus fat tissue between the tumor and the renal parenchyma.

**Fig. 3:**
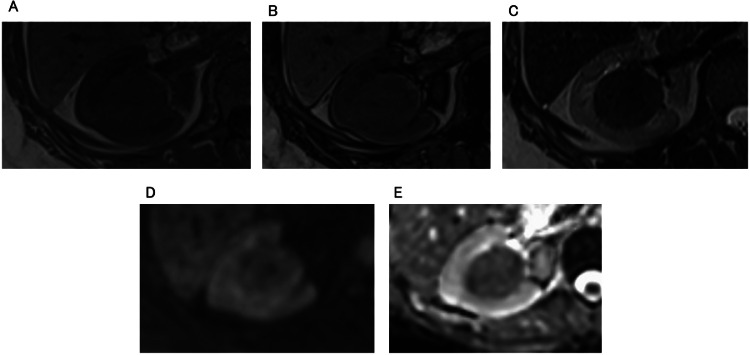
MRI. (A) T1 in-phase images, (B) T1 out-of-phase images, (C) T2WI, (D) diffusion-weighted image (DWI), and (E) apparent diffusion coefficient (ADC) map. Tumor demonstrates a homogeneous low signal on T1W (A). There was no low signal indicative of a small amount of fat on T1 out-of-phase images (B). On T2WI, a homogeneous low signal is observed (C). ADC value is 1.27 × 10^-3^ mm^2^/sec, demonstrating no significant diffusion restriction (D, E).

MRI was performed (Fig. 3). The tumor exhibited a homogeneous low signal on T1 in-phase images, with no high signal indicative of hemorrhage. No intravoxel fat was detected on T1 out-of-phase images. On T2-weighted images (T2WI), the lesion displayed homogeneous low signal. No significant diffusion restriction was observed.

The tumor was located in the renal sinus with well-defined borders. On dynamic contrast-enhanced CT, it demonstrates a gradual increase in homogeneous enhancement, while MRI revealed a homogeneous low signal on T2WI. Small fat tissue, compressed renal calyces, and vessels were observed between the tumor and renal parenchyma, making it extremely difficult to determine whether the tumor originated in the renal parenchyma or the renal sinus. If the lesion originated from the renal parenchyma, papillary renal cell carcinoma or fat-poor angiomyolipoma (AML) were considered likely diagnoses. These conditions are common and consistent with the findings on both dynamic contrast-enhanced CT and MRI. The absence of diffusion restriction was a finding consistent with fat-poor AML rather than papillary renal carcinoma. This is because typical papillary renal carcinoma demonstrates diffusion restriction. However, some papillary renal carcinomas also had unclear diffusion restriction, making differential diagnosis challenging. Conversely, if the lesion originated from the renal sinus, a solitary fibrous tumor (SFT) was considered. However, SFT is a rare neoplasm that typically presents as a hypervascular tumor, and the CT findings in this case were inconsistent with such characteristics. Unfortunately, a CT-guided biopsy could not be performed because of the absence of a safe puncture route. Given the possibility of papillary renal cell carcinoma, a nephrectomy was performed.

**Fig. 4:**
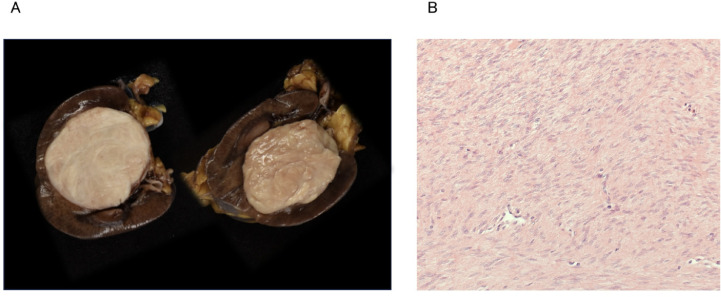
Resected specimen (A), Hematoxylin and eosin stain (B). Macroscopic examination revealed a well-circumscribed white tumor measuring 4.3 cm × 4 cm, revealing no continuity with the renal parenchyma (A). Non-atypical smooth muscle cells proliferated densely with no evidence of hemorrhage or necrosis (B).

Macroscopic examination revealed a well-circumscribed white mass with no continuity with the renal parenchyma, indicating that the tumor originated from the renal sinus (Fig. 4). Histopathological examination demonstrated a dense proliferation of smooth muscle cells without atypia (Fig. 4). No hemorrhage, necrosis, or fat was observed within the tumor. Furthermore, the tumor was found to be in contact with a branch of the renal vein. Based on these findings, the final diagnosis was a leiomyoma arising from the renal sinus vein.

## Discussion

Renal leiomyomas and renal cell carcinomas (RCCs) exhibit distinct epidemiological characteristics. Renal leiomyomas predominantly occur in females in their 40s and 50s [1], whereas RCCs are more common in males in their 50s and 70s. The present case involved a woman in her 30s, and retrospective analysis indicated that the features were more consistent with renal leiomyomas than with RCCs. However, RCCs in patients aged 39 years or younger accounts for 3.4% of all RCCs [4]. In other words, although uncommon, RCCs can occur in individuals in their 30s. Therefore, RCCs cannot be ruled out solely on the basis of epidemiological factors, highlighting the importance of imaging-based diagnosis.

CT findings of renal leiomyomas typically reveal well-defined, relatively homogeneous masses [5,6]. On plain CT, these lesions often exhibit heterogeneous or slight hyperdensity compared with the renal parenchyma [5,6]. On contrast-enhanced CT, they demonstrate lower enhancement than the surrounding renal parenchyma [5,6]. MRI findings of renal leiomyomas without degeneration demonstrate a homogeneous isosignal on T1WI and a low signal on T2WI. When edema, calcification, or cystic degeneration occurs, the tumor signal becomes heterogeneous [[7], [8], [9]]. Therefore, renal leiomyomas without degeneration, such as in this case, may present as slightly hyperdense relative to the renal parenchyma on plain CT, exhibit weaker enhancement on dynamic contrast-enhanced CT, and demonstrate a uniform low signal on T2-weighted MRI. These imaging characteristics closely resemble those of papillary RCCs without hemorrhage, making radiologic differentiation challenging. Consequently, the primary point for differential diagnosis is the estimated location of origin.

Renal leiomyomas most commonly originate from the renal capsule, pelvis, calyces, or the blood vessels surrounding the kidney [6]. These tumors typically arise from the subcapsular or capsular region (90%) and are rarely located within the renal sinus (10%) [3]. Unlike papillary RCCs, which originates in the renal cortex, renal leiomyomas develop near the surface of the kidney, specifically along the boundary between the renal parenchyma and the surrounding adipose tissue. Literature review revealed that renal leiomyomas are usually contiguous with the kidney or cause external compression of it, while those completely involving the renal parenchyma are rare [[5], [6], [7],[10], [11], [12]]. In the present case, the tumor originated from the renal sinus vein, an uncommon site for renal leiomyomas. Similar to tumors arising from the renal capsule, it was located adjacent to the kidney and exerted compressive effects on the renal parenchyma and calyces from outside the parenchyma. The fine layer of fat observed on CT imaging between the tumor and the renal parenchyma supported a renal sinus origin. Similar findings have also been reported in a schwannoma that developed within the renal sinus [13].

A biopsy is essential for the definitive diagnosis of renal leiomyoma. In this case, the tumor originated from the renal sinus, indicating that it was surrounded by renal parenchyma. Consequently, no safe puncture pathway was available, making CT-guided biopsy challenging. If a biopsy had been possible, the diagnosis of renal leiomyoma might have been confirmed. Most renal leiomyomas originate from the renal capsule [3]. When renal leiomyoma is suspected, a biopsy should be considered to prevent unnecessary surgical intervention.

In young women presenting with tumors potentially originating in the renal sinus, particularly when contrast-enhanced CT demonstrates poor enhancement and T2-weighted MRI reveals low signal intensity, renal leiomyomas must be included in the differential diagnosis. The key to differentiating papillary RCCs on imaging lies in its epidemiological characteristics and the presumed site of origin.

## Patient consent

Verbal informed consent for publication of this case report and accompanying images was obtained from the patient via telephone, and this consent was documented in the patient’s medical record.
